# Editorial

**Published:** 1863-10

**Authors:** 


### Editorial.

A TREATISE ON HYGIENE: WITH SPECIAL REFERENCE TO THE MILITARY SERVICE. By WILLIAM A. HAMMOND, M.D., Surgeon-General of U. S. Army; Fellow of the College of Physicians of Philadelphia, &c., &c., &c., &c. Philadelphia: J. B. LIPPINCOTT & Co. 1863.

This is a full-sized octavo volume of 604 pages, published in excellent style, and containing a treatise on a very important branch of medical science, by one who occupies a high official position. After a short preface and introduction, the work is divided into three sections, as follows:—

Section 1st.—On the examination of recruits.

Section 2d.—Of the agents inherent in the organism which affect the hygienic condition of man.

Section 3d.—Of agents external to the organism which act upon the health of man.

The work is illustrated by 74 cuts, representing chiefly plans for hospitals and apparatus connected therewith. The first section, which occupies about forty pages, we shall pass by with the single remark, that it contains plain, practical, and generally judicious directions in relation to the examination of recruits: pointing out plainly the items of qualification and disqualification to be observed by the medical examiner. The next section contains ten chapters on the following subjects:—1. Race; 2. Temperaments in general; 3. Particular temperaments; 4. Idiosyncrasy; 5. Age; 6. Sex; 7. Hereditary tendency; 8.

Habit; 9. Morbid habits; 10. Constitution. These ten chapters simply present a brief summary of such facts and opinions relating to the several topics as are familiar to every reading man in the profession. Together, they occupy eighty-six pages of the work.

Section III. contains twenty-nine chapters as follows:—1. The atmosphere; 2. The accidental or non-essential constituents of the atmosphere; 3. Physical properties of the atmosphere; 4. Temperature; 5. Light; 6. Electricity; 7. Water; 8. Soil; 9. Locality; 10. Climate; 11. Acclimation; 12. Habitations; 13. Hospitals; 14. Principles of hospital construction; Field hospitals; 16. Lighting of hospitals; 17. Heating of hospitals; 18. Ventilation of hospitals; 19. Barracks; 20. Camps; 21. Food; 22. Alimentary principles; 23. Physiological and sanitary relations of food; 24. Animal compound aliments; 25. Vegetable compound aliments; 26. Accessory food; 27. Alimentation of the soldier; 28. Clothing; 29. The hygienic relations of clothing with the several parts of the body.

It will be seen that six of these chapters, filling more than one hundred and forty pages, are devoted to the consideration of hospitals and their management, and constituting, by far, the most valuable part of the work. The remaining chapters, except the 26th, like those in the second section, present only brief summaries of the most common-place and familiar facts in relation to each topic. For instance, in the chapter on Temperature, instead of a philosophical explanation of the effects of caloric on living animal structures, and the best means of obviating the consequences of its excess or deficiency, we have reproduced the old and familiar story of the excursion of "Sir Joseph Banks, Dr. Solander, and nine others;" and the usual allusion to the destruction of Napoleon's army on the frozen plains of Russia. We are also told that the direct rays of the sun, in hot weather, are liable to produce "sun-stroke," and that a wet cloth worn on top of the head is a good prophylactic; but in what "sun-stroke" consists, pathologically, is not so much as hinted at. The only chapter in which the author attempts to enter upon a philosophical discussion of the subject under consideration, is that which treats of "Accessory Food." Under this head, the author includes condiments, such as pepper, mustard, vinegar; drinks,—as alcohol and its compounds; tea and coffee; and tobacco. The comments on the three first articles are brief and unexceptional. Those in relation to alcohol and its compounds are of a widely different character. He introduces the subject with the following very modest declaration, namely:—"The chief reason why the advocates of a total prohibition of the employment of alcoholic liquors have been unable to carry conviction to those to whom they have addressed themselves is, that their remarks have *mainly consisted of invectives*, and that whatever facts they have brought forward have been altogether based upon the *immoderate* use of the agents in question." For fifty years, this has been the uniform reply of the advocates of alcoholic liquors as beverages, to all arguments, whether founded on statistical facts, showing the relative power of physical endurance between those who use and those who do not use alcohol; the relative ratio of sickness and mortality; or on direct physiological experiments. To show by actual results of labor in every department of human toil, whether in the harvest-field, the workshop, the brickyard, the army, or the navy; whether in summer or in winter; whether under the burning rays of a torrid sun, or amidst the icebergs of the arctic regions; that those who use alcoholic beverages, whether fermented or distilled, actually do a less average amount of labor, are capable of less physical endurance, and suffer a higher ratio of attacks of sickness than those who, under exactly the same circumstances, wholly abstain from such liquors, is to deal *mainly* in "invectives," is it? To prove, by direct experiment, that alcohol enters the blood unchanged, disturbs and perverts the sensibility and action of the nervous structures, depresses the elementary properties of all the tissues,—thereby retarding organic changes,—and disturbing the natural play of those affinities, by which nutrition, disintegration, and secretion are effected; and is finally evolved again from the economy as alcohol, from the lungs, kidneys, &c., is also dealing mainly in "invectives," we suppose.

To show by the physiological action of alcohol, and by innumerable cases, taken from every rank of human society, that the habitual *moderate* use of alcoholic drinks leads, in nine cases out of every ten, to the "immoderate" use of the same, is also dealing in "invectives." Well, be it so for the present. Immediately following the paragraph quoted above, our author proceeds as follows:—"No one can for a moment deny that alcoholic liquors, when used in excessive amount, are not only injurious to the individual, but are also in the highest degree pernicious to society. * * * But are such facts to influence us against the *proper* use of all beverages which contain alcohol. * * * Do we reject mutton because some one has killed himself by eating too heartily of mutton-chops?"

We certainly should not condemn the proper use of an article merely because its abuse produced injurious effects. But we cannot help asking, whether the W. A. Hammond, M.D., author of the work before us, ever heard of a certain Order, No. 6, excluding calomel from the supply-table of the army, *because it had been used* "immoderately" by some of the army surgeons, issued by W. A. Hammond, M.D., Surgeon-General of United States Army?

Without wasting words, however, on minor matters, let us first see what are the actual effects of alcohol on the human system, as shown by the experiments and researches of Drs. Percy, Prout, Böcker, Lellemand, Hammond himself, and others, as collated in the chapter under consideration. They may be summed up as follows:—

1st.—The alcohol taken into the stomach is rapidly absorbed into the blood, circulated with it throughout the whole system, and is eliminated chiefly through the lungs and kidneys; being readily detected, by the proper tests, both in the vapor of the breath and in the urine.

2d.—While in the blood, it produces an exhilerating effect upon the brain and nervous centres, causing thereby disturbance in the mental operations and sensibilities of the patient.

3d.—Its presence in the blood diminishes the aggregate amount of eliminations from the several excretory organs of the body, doubtless by diminishing both structural disintegration and secretion.

These propositions may be considered as well settled by a great variety of experiments and observations, both in Europe and America. But the practical inferences to be drawn from them are still the subjects of much controversy. Thus, a class of chemico-physiologists, embracing LIEBIG, MOLESCHOTT, HAMMOND—the author of the work before us, and many others, claim that, because the presence of alcohol in the human system diminishes the aggregate amount of eliminations, and causes an increase in the weight of the body, provided the digestion of other food goes on as usual, it actually supplies the place of food.

Thus, on page 539 of the work before us, Dr. HAMMOND says:—"We have seen that it takes the place of food, and that the weight of the body increases under its use. Any substance which produces the effects which we have seen to attend on the use of alcohol, even though it is not demonstrable at present that it undergoes convertion into tissue, is food;" and on the next page we find a quotation from MOLESCHOTT, as follows:—"Alcohol is a savings-bank for the tissues,—if the expression will be understood. He who eats little and drinks moderately of alcohol, retains as much in his blood and tissues as he who, in corresponding relations, eats more and drinks neither beer, nor wine, nor brandy." It is on this assumption, that alcohol is a substitute for food, that our author and others of the same physiological school base nearly all their reasoning in favor of the general use of alcoholic compounds as beverages. It is mainly on this same assumption that a very large class of medical writers and practitioners base their recommendation of alcoholic drinks in the treatment of numerous important diseases. Hence the question, whether this assumption or inference is correct; whether it legitimately follows from the premises or facts proved, is one of the highest importance in its relations to physiology, therapeutics, and social life. Plainly and concisely stated, the premises and the inference is as follows:—

1st.—It is definitely proved, by a great variety of experiments, that under the influence of alcohol, other things being equal, the sum total of the excretions or eliminations from the lungs, skin, kidneys, bowels, &c., are diminished, and the body gains in weight. This is the premise from which it is inferred.

2d.—That such diminution of elimination is caused by the alcohol retarding the natural disintegration of the tissues, while the processes of assimilation and construction of tissue is allowed to continue; and that thereby the alcohol acts the part of actual food.

Thus, the naked question is evolved: whether a retardation of the natural disintegration of tissue and elimination of the resulting effete matter is actually equivalent to, or will, physiologically, compensate for, a certain amount of assimilation and nutrition? Dr. HAMMOND, and the advocates of the use of alcoholic beverages, generally assume the affirmative; but is their position in consonance with the known and acknowledged laws which govern the nutrition and disintegration of living tissues? It is universally conceded, that living organized animal structures are composed of organic atoms or cells, none of which remain permanent, but each of which serves its purpose and gives place to a new one. It is further generally conceded, that the performance of every functional act or display of force, whether mental or physical, is attended by more or less displacement of these atoms. It is this constant displacement of old organic atoms that renders a regular supply of food or ingesta necessary to the maintainance of life in all the higher orders of animals. This constant atomic change, being a physiological law of the organization, taking place under the guidance of an inherent vital affinity, cannot be retarded, except by directly impairing or weakening the affinity itself, or by introducing some new agent possessing a stronger affinity for some of the atoms composing the tissues than is possessed by oxygen or whatever naturally effects the primary steps of disintegration. To do either of these, manifestly induces a *pathological* condition incompatible with the continuance of health. For every intelligent physiologist knows, that

34A

on the constant display of vital affinity in the organic or atomic changes taking place in the tissues, depends the development of caloric to maintain animal temperature; the generation of nerve sensibility; the elaboration of the secretions; and, indeed, all the distinctive phenomena of animal life. Hence, whatever agent introduced into the blood, in a healthy state of the system, is capable of retarding the process of disintegration, must, if persisted in, necessarily produce either disease, or perverted nutrition, or both. Dr. HAMMOND himself sees, at least partially, this conclusion, as is evident from the following, from page 540:—"Alcohol retards the destruction of tissue. By this destruction force is generated, muscles contract, thoughts are developed, organs secrete and excrete. Food supplies the material for new tissue. *Now, as alcohol stops the full tide of this decay, it is very plain that it must furnish the force which is developed after it is ingested. How it does this is not clear.*"

Here is a full acknowledgment of the fact: that to retard the metamorphosis or disintegration of the tissues is to retard, in the same ratio, all the force producing processes or organic functions of animal life. True, he attempts to escape from the dilemma by supposing that the alcohol itself comes to supply the force which its presence prevents the natural atomic changes in the tissues from generating; but how it does this, he frankly confesses, "is not clear."

His attempt, on the next page, to explain the matter by saying, "it is not at all improbable that alcohol itself furnishes the *force* directly, by entering into combination with the products of tissue decay, whereby they are again formed into tissue, without being excreted as urea, uric acid, &c.;" is not only destitute of proof, but, unfortunately, in direct conflict with the results of a well-devised series of experiments by LALLEMAND, PERRIN, and DUROY, which show that all the alcohol taken, is ultimately eliminated through the excretory organs unchanged. The same is confirmed by Dr. HAMMOND'S own experiments, during which the alcohol was generally detected both in the breath and the urine some time after it was absorbed from the stomach.

Thus, turn whichever way they will, those who advocate the doctrine, that a retardation of disintegration is equivalent to nutrition, involve themselves in difficulty. Indeed, the proposition itself is a physiological absurdity. If it were true, it would only be necessary to find some substance that would arrest the processes of tissue disintegration entirely, and we might live on without the necessity or *expense* of eating at all. Indeed, if alcohol is capable of retarding tissue destruction, and, at the same time, of furnishing the required *force* itself, what is to hinder a man from living on it indefinitely? Whether fascinated by the beautiful theory of HAMMOND or not, many a poor fellow has practically tested it, but has generally been unfortunate enough to have the experiment cut short by a fit of *delirium tremens*. We have thus far pursued the subject as though the experiments cited by our author actually proved that alcohol retarded the disintegration of tissues; but they really prove no such thing. They simply show, that, while alcohol is present in the system, the sum total of eliminations is diminished and the weight of the body increased, provided the usual supply of ordinary food has been continued. Whether the diminished excretions are owing to retarded disintegration of tissues or to the direct action of alcohol on the excretory organs, whereby their power to perform their respective functions becomes impaired, is a question open for discussion; and one to which we may recur at some future time.

The only remaining ground on which alcohol can be claimed as food, in any sense, is that originally put forth by LIEBIG, and quoted approvingly by our author, namely, that, like other hydro-carbonaceous substances, it furnishes material for respiration. This, however, is also directly disproved by all the experiments cited in the chapter before us; by a series of experiments performed by myself, and reported to the Annual Meeting of the American Medical Association, in 1851; and by a great variety of facts derived from observation. It is evident that alcohol can act as respiratory food only by entering into combination with the oxygen; the resulting products of which would be carbonic acid gas and water; and, as a consequence, we should have an abundance of carbonic acid exhaled from the lungs and an increased temperature of the system. Whereas the experiments cited by our author all show a diminution of carbonic acid in the exhaled air and the presence of alcohol unchanged; while those reported by myself, show a positive decrease of temperature while under the influence of that agent. There are several other items in this chapter we had intended to notice, but time will not permit at present. We have written enough to show the utter fallacy of the main positions of the author; in his effort to convert what he acknowledges in the outset to be a "violent poison," into beneficial food and drink. That part of the chapter commending the use of tobacco, is founded on the same fallacies and exhibits the same absurdities.

Indeed, his direct definition of food, necessarily destroys all distinction between aliments and poisons,—between food and medicine. After stating that the presence of alcohol in the system diminishes the destruction of tissue and causes the body to increase in weight, he says:—"Any substance which produces the effects which we have seen to attend on the use of alcohol, even though it is not demonstrable at present that it undergoes conversion into tissue, *is food.*" Now, it is well known that opium, and, probably, all the other narcotics, diminish the organic changes; and, if taken in moderate doses, allowing appetite and digestion to continue, the body increases in weight. The same results have been known to accompany the use of arsenic and other acknowledged poisons. Would Dr. HAMMOND, therefore, call opium and arsenic food? To do so would only be equalled in absurdity by the following remark of LIEBIG, gravely quoted as authority by our author:—"The use of spirits is not the cause, but an *effect of poverty.* It is an exception to the rule when a *well-fed* man becomes a spirit-drinker." Such an assertion, in this country, where a loaf of bread costs but a trifle more than a glass of whiskey or a mug of beer, and where men are almost as frequently seen staggering in broad-cloth as in rags, will scarcely produce any other effect than to excite a smile at the theoretical vagaries of men eminent in some departments of science. It is hardly necessary to say, in conclusion, that the examination of this work of Dr. HAMMOND has exceedingly disappointed us. The publishers have done their part of the work well; but, so far as it claims to be a treatise on hygiene, it is very superficial and abounding in most mischievous errors.

